# Interplay of multiple synaptic plasticity features in filamentary memristive devices for neuromorphic computing

**DOI:** 10.1038/srep39216

**Published:** 2016-12-16

**Authors:** Selina La Barbera, Adrien F. Vincent, Dominique Vuillaume, Damien Querlioz, Fabien Alibart

**Affiliations:** 1 Institut of Electronic, Microelectronic and Nanoelectronic, CNRS, boulevard Poincarré CS 60069, 59652 Villeneuve d’Ascq, France; 2 Centre de Nanosciences et de Nanotechnologies, CNRS, Univ. Paris-Sud, Université Paris-Saclay, C2N - Orsay, 91405 Orsay cedex, France

## Abstract

Bio-inspired computing represents today a major challenge at different levels ranging from material science for the design of innovative devices and circuits to computer science for the understanding of the key features required for processing of natural data. In this paper, we propose a detail analysis of resistive switching dynamics in electrochemical metallization cells for synaptic plasticity implementation. We show how filament stability associated to joule effect during switching can be used to emulate key synaptic features such as short term to long term plasticity transition and spike timing dependent plasticity. Furthermore, an interplay between these different synaptic features is demonstrated for object motion detection in a spike-based neuromorphic circuit. System level simulation presents robust learning and promising synaptic operation paving the way to complex bio-inspired computing systems composed of innovative memory devices.

Bio-inspired computing represents today a major challenge at different levels ranging from material science, design of innovative devices and circuits to computer science. In particular, it is highly attractive to identify materials which possess multiple features on different timescales, to emulate features seen in the brain. In this work, we propose a detailed analysis of resistive switching dynamics in electrochemical metallization cells for synaptic plasticity implementation. We show how filament stability associated to Joule effect during switching can be used to imitate key synaptic features such as short term to long term plasticity transition and spike timing dependent plasticity. Furthermore, from a computing point of view, we show how the interplay between these different synaptic features can be harnessed for video processing in a spike-based neuromorphic circuit. Our system level simulations present robust learning and promising synaptic operation, paving the way to complex bio-inspired computing systems composed of innovative memory devices.

Considerable research is now looking at developing bioinspired computing systems that would approach the brain performances in terms of low-power computing and versatility^1^,^2^. Impressive milestones have been reach in this direction, but most demonstrated brain-inspired systems rely on purely-CMOS solutions and lack a scalable implementation of synapses, the connections between neurons^1^,^3^,^4^. Additionally, in CMOS implementations, it is extremely expensive to provide synapses with “plasticity” features, the key mechanism for learning. Emerging technologies based on specific materials, along the concept of memristor^5^,^6^, provide a solution for compact implementation of synapses and are therefore a critical element for the success of bioinspired electronics^1^,^2^,^7^,^8^. Until now, such devices have been developed along two distinct directions. From one hand, it is attractive to look for simple but ultra-high density synaptic memory. Such research can capitalize on the industrial development of resistive random access memory^9^,^10^ but does not provide an easy way to implement plasticity. On the other hand, a more forward-thinking “biomimetic” approach aims at providing feature-rich memories that replicate and implement plasticity features directly^11^,^12^,^13^,^14^,^15^. Some proposals with memristive devices have successfully implemented synaptic plasticity features such as short term plasticity (STP)^11^,^16^,^17^. STP was implemented by taking advantage of the memory device volatility which tends to relax toward its stable state on short time scale (from millisecond to second) after potentiation (i.e. increase of conductance) or depression (i.e. decrease of conductance). However, isolated short term plasticity has limited computational value, and can be used only for simple tasks^18^,^19^. A more advanced plasticity feature observed in several devices^12^,^13^,^20^ corresponds to the transition between short term plasticity and long term plasticity (LTP). A weak potentiation results in conductance relaxation on short time scale (equivalent to STP) while a stronger potentiation results in slower relaxation (or even absence of relaxation) associated to LTP. This effect was observed when relaxation of conductance (i.e. volatility) was dependent on the conductance state reach after potentiation. However, if this property can be reminiscent of the concept of memory consolidation, it remains limited in terms of practical applications. On the other hand, Hebbian-like synaptic plasticity is considered as the foundation for the development of complex computing functions. In this case, pre- and post-neuron activity correlation defines the synaptic change. One of the most typical Hebbian-like learning mechanisms is the Spike Timing Dependent Plasticity (STDP). It consists in a modification of the synaptic conductance depending on the spike timing (i.e. time correlation) of both pre and post neurons to which a synapse is connected^21^. In most proposals with memristive devices, STDP is implemented with sophisticated overlapping programming pulses which artificially implement the time distance between the pre and post pulses^2^,^14^,^15^,^22^,^23^. Only few materials display intrinsic physics where internal switching dynamics lead to STDP characteristics^24^,^25^. In this approaches, time correlation is ensured by time lag effects after a switching event encoding the time distance between pre and post events. Since these approaches do not required a specific and complex pulse design,it reduces significantly the complexity of the overall circuit and offers promising strategies for bio-realistic implementation of neuronal circuit with nanodevices. Nevertheless, STDP models only a small fraction of the behaviors of biological synapses. Coupling STDP with other plasticity effects has been investigated in ref. 26 and would improve the computational capability of bio-inspired neuronal systems. This is what we address in the present paper. We identify experimentally a material providing intrinsic STDP as well as other plasticity mechanisms, and investigate by computer simulation how this can be harnessed for computing.

Among the large panel of emerging memory technologies, we focus on Electro-Chemical Metallization (ECM) cells^27^ based on Ag_2_S ionic conductor. ECM cells have demonstrated state of the art performances for memory applications^28^, but most interestingly they provide dynamical behaviors that allow bio-inspired features implementation such as Short Term and Long Term plasticity^12^. Through this article, we propose a detailed analysis of the switching dynamics in filamentary switching ECM cells, combining filament growth and relaxation with second order effect such as Joule heating during switching. We show that these two physical mechanisms can lead to an interplay between short term to long term memory transition and STDP behavior. We proposed a bio-inspired model that can qualitatively describe the different plastic features. By the means of system-level simulations based on the proposed model, we finally show how an interplay of these different synaptic features can be used for dynamic motion learning, for a proof-of-concept learning application.

## Results and Discussion

### Short term to long term plasticity

**Figure 1:**
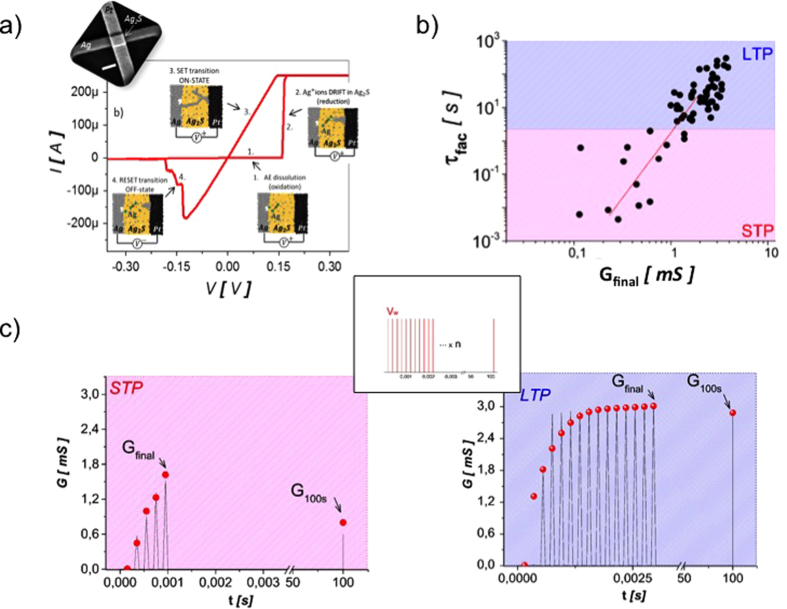
(**a**) Bipolar switching characteristic of ECM cells with conventional hysteresis loops (inset: SEM image of the device in a crosspoint configuration. Scale bar is 200 nm). (**b**) Evolution of the characteristic time constant τ_fac_ as a function of final conductance G_final_ reached at the end of a potentiation experiment for various setup (various pulse number from 3 to 150 and frequencies from 1 to 5 kHz). Red line is a power law fit of the measurements. (**c**) Examples of potentiation experiments with STP (left) and LTP (right) characteristics (black lines) fitted by the iterative model V1 (red circles).

Filamentary memristive devices were fabricated in a cross-point configuration of 200 × 200 nm^2^ with Ti/Pt bottom electrode, Ag_2_S ionic conductor and Ag top electrode (inset Fig. 1a).The basic switching mechanism during SET (ON transition) is based on the oxidation of Ag into Ag^+^ at the top electrode, reduction of Ag^+^ ions into conductive Ag filaments across the ionic conductor while RESET (OFF transition) corresponds to Ag oxidation from the filaments and reduction to the top electrode. Such reversible switching effect presents bipolar switching characteristics (Fig. 1a). We previously reported a detailed analysis of filament stability^20^ leading to the implementation of short term to long term transition, which we review briefly in this section. Simple positive square shape voltage pulses applied to the memory device induce a potentiation associated to the growth of the metallic filament. A small number of programming pulses leads to thin filaments that tend to dissolve quickly without stimulation and implements STP effects while a large number of programming pulses leads to large filaments that are stable on long time scale without stimulation and implements LTP effects (Fig. 1c). Filament stability was associated to a competition between surface and volume energy in the metallic filament with unstable thin filaments (maximization of surface energy) and stable large filaments (maximization of volume energy). The stability of the filament can be conveniently described by analyzing the characteristic time constant of relaxation τ_fac_ after a potentiation (supplementary material, Figure S1). Figure 1b plots the characteristic relaxation time constant for various potentiation experiments (i.e. different number of pulses and frequency from 1 to 5 kHz) with different final conductance states G_final_ reach after potentiation. The relationship between τ_fac_ and G_final_ presented in Fig. 1b was fitted with a power law function:


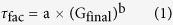


with a = 3.40 × 10^12^ s/S^b^ and b = 4.

In order to describe the device conductance evolution under simple positive square shaped pulses, we developed an iterative model inspired by the phenomenological model developed in ref. 29 for biological synapses. This model (named “model V1” in the following) describes the balance between conductance relaxation when the memory device is not stimulated and the increase of conductance induced by positive pulses. Starting from any conductance state G_n−1_ after the n − 1^th^ pulse with a value between G_min_ (minimal conductance fixed at 1 μS for our devices) and A_0_ (maximum conductance that can be reached by our devices), the device conductance relaxes over time following:


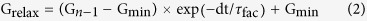


With τ_fac_ evolution described according to equation (1) by:


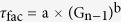


Any n^th^ positive pulse will induce a potentiation toward A_0_ set by:


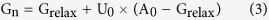


With U_0_ a constant between 0 and 1 associated to the amount of potentiation induced by a single pulse.

Model V1 describes conveniently the conductance response of our devices to pre-neuron pulses of fixed amplitude and width (Fig. 1c) separated by a time interval dt. The model parameters A_0_ and U_0_ which describe the potentiation have been observed to remain constant for dt > 200 μs (i.e. maximal frequency of 5 kHz) and provided a good quantitative modeling in this frequency range.

This model grabs the main properties of our devices that will present key features of interest for synaptic plasticity implementation. (i) In absence of stimulation, natural relaxation of the filament will tend to set all weak synaptic weights to their minimum value on short time scale while only strong synapses with long time constant τ_fac_ will stay in their potentiated state. This behavior has been observed in biological systems where weak synapses tends to disappear over time^30^ and is reminiscent of weight penalty strategies used in machine learning approaches^31^. (ii) Potentiation of the synaptic weights is multiplicative (through the U_0_ parameter) and will lead to bounded synaptic weights thus cancelling weight divergence. However, these features remain limited in terms of computing capability without additional long term effects, and more precisely without Hebbian effects. It should be noted that the boundary’s definition between short term and long term is a task dependent issue. In fact, the mean duration of an event to be learnt should correspond to the short term regime in order to observe a transition toward long term regime after repetition of the event.

### STDP-induced short term to long term plasticity transition

The plasticity mechanism reported in the previous section can be qualified as “non-Hebbian” (i.e. resulting from pre-only or post-only synaptic events). “Hebbian-type” learning, and in particular STDP, is defined as a correlation between pre and post synaptic events. In biological systems, since synapses are not bidirectional, pre/post events are unequivocally defined at the synaptic connection level. For example, pre synaptic events lead to neurotransmitter release while post synaptic events modify Ca^2+^ concentration and the time correlation between these two different signals define STDP events. In our case, since electronic synapses are bidirectional and pre/post events are equivalently defined as a spike of voltage, STDP needs to be defined at the system level. Time distance between two consecutive pre (or post) synaptic events can be constrained to be larger than a minimal time interval ΔT (this can be imposed by setting a given refractory period at the neuron level). We can conveniently define STDP events (i.e. pre/post interaction) by considering the time window below ΔT. Any pair of spikes with time interval dt < ΔT will result from time correlation between a pre and post synaptic event. In the present section, we show experimentally that ECM cells, in addition to the non Hebbian learning reported in the previous section, also intrinsically exhibit a Hebbian form of plasticity: they implements a Hebbian STDP corresponding to an increase of the synaptic weight when time correlation between pre- and post-neuron firing is experienced by the synapse. While this STDP function do not implement the causality between pre and post events, it is reminiscent of biological STDP observed in GABA-ergic neurons in hippocampal culture^32^.

**Figure 2:**
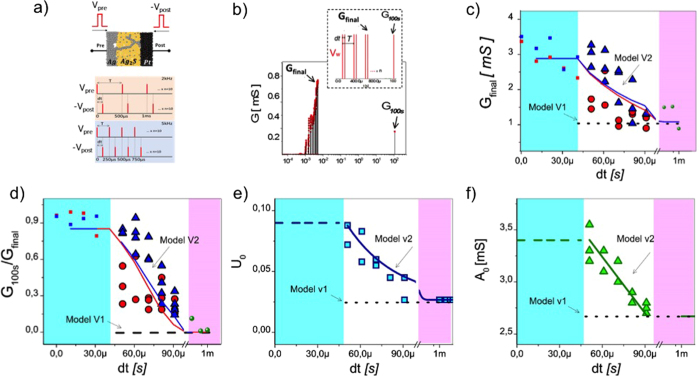
(**a**) STDP protocol. 10 pre and post pulses pairs are applied across the ECM cell with various dt between pre and post and two mean frequencies < f > defined as 1/T. Vpre was 400 mV and Vpost was −400 mV (**b**) STDP measurement. A pair-based burst of pulses induces a potentiation of the synaptic device to Gfinal. The relaxation is estimated by measuring the conductance after 100 s without any stimulation. (**c**) Evolution of the potentiation after a STDP protocol as a function of dt. Pink region: control experiment without pair-pulses (i.e. pre neuron only potentiation). White region: STDP-induced potentiation with non-overlapping pulses. Blue region: potentiation with pulse overlapping. The black dashed line corresponds to the simulated results of the STDP protocol with model V1. Red and Blue lines correspond to the simulated results based on model V2 for STDP pairs at 2 kHz and 5 kHz, respectively. (**d**) Evaluation of the LTP characteristic induces by STDP protocol. (**e**) and (**f**) Model parameters A_0_ and U_0_ used for fitting Gfinal and G_100s_/G_final_ during STDP experiments with the iterative model.

In order to evaluate the potentiation induced by a pair of pre and post pulses with equivalent shape, we developed a bio-inspired STDP protocol (Fig. 2a). The spikes used for this protocol were simple square-shaped pre (post) pulses of 50 μs width and 400 mV (−400 mV) amplitude, respectively. Two parameters were tuned during the STDP experiment: (i) the time correlation between pre- and post-pulses dt and (ii) the mean frequency < f > of pre-neuron firing associated to a period ΔT. All the experiments started from a low conductance state of the ECM cell. G_final_ corresponds to the final conductance state at the end of the STDP protocol. After each STDP protocol, long term plasticity -induction was evaluated by applying a single pre-pulse after 100 s of rest and measuring the conductance G_100s_ (Fig. 2b). Figure 2c and d present the STDP results obtained for our ECM crosspoint devices. A clear increase of potentiation (i.e. conductance) from 1 mS to 3.5 mS and long term plasticity induction (increase of G_100s_/G_final_ from 0 to 1) is measured for time correlation between pulses smaller than 100 μs. In addition, when dt is decreased toward 50 μs, this effect is strengthened, therefore reproducing a gradual STDP windows as observed in biology. Smaller time correlation with dt smaller than 50 μs induced pre- and post-pulse overlapping (pulse width was 50 μs). Since large voltages (i.e. 2 × V_pulse_) are obtained in this case, fully potentiated weights in their long term plasticity regime (G_100s_/G_final_ ≈ 1) were measured (red and blue squared dots in blue region, Fig. 2c and d). Control experiments with pre-neuron spikes only were performed and showed weak potentiation (G_final_ = 1 mS) and no long term plasticity (G_100s_/G_max_ ≪ 1) corresponding to short term plasticity regime – Fig. 2c and d, green dots in pink region.

A first comparison of the STDP measurements with model V1 can be realized in order to extract the origin of STDP in ECM devices. We calculated the expected G_final_ and G_100s_/G_final_ values for similar spike protocol with model V1 for non-overlapping pulses. As presented in Fig. 2c and d, model V1 failed at reproducing the STDP measurements. As pre- and post-pulses are equivalent, a first conclusion is that short time scale interactions between two successive pulses is not captured by model V1. Modification of model V1 into model V2 was realized by setting U_0_ and A_0_ as free parameters for fitting the STDP measurements. Figure 2e and f present the evolution of U_0_ (amount of potentiation induce by a pulse) and A_0_ (maximal conductance that can be reach by a fully potentiated synapse) as a function of time correlation dt for non-overlapping pulses during STDP measurements for 2 and 5 kHz mean frequency stimulation. For large dt (i.e. dt > 90 μs), U_0_ and A_0_ presented similar values as the one extracted from control experiment and pre-neuron only excitations (U_0_ = 0.0267 and A_0_ = 2.7 mS, pink region in Fig. 2e and f), corresponding to model V1. For 50 μs < dt < 100 μs, STDP experiments show an increase of potentiation (G_final_) and a clear transition toward long term potentiation (G_100s_/G_final_ → 1). Fitting of this behavior is possible by increasing both U_0_ and A_0_ when dt is decreased. In other words, the increase of Gfinal and G_100s_/G_final_ is captured by an increase of U_0_ and A_0_. For dt < 50 μs, A_0_ and U_0_ were assigned to a saturated value corresponding to pre and post pulse overlapping and to fully potentiated weights. We described U_0_ and A_0_ evolution with exponential decay and with linear fitting in the short dt regime, respectively:


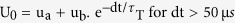



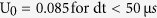



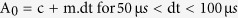



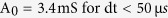



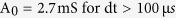


where fitting parameters are: u_a_ = 26.7 × 10^−3^, u_b_ = 0.2717, τ_T_ = 34.1 μs, c = 4.32 mS and m = −18 S/s. These two relationships were introduced into model V1. Thus, the conductance evolution after application of a pulse (either pre or post) can be described by equation (2) and (3) with addition of A_0_ and U_0_ functions.

The resulting model provides a qualitative description of long term plasticity induction and potentiation during STDP measurements, as shown by the red (2 kHz) and blue (5 kHz) lines in Fig. 2d and e. Model V2 does not discriminate the 2 kHz and 5 kHz STDP experiments while measurements indicate a quantitative difference of trend between the two pre-neuron spiking frequencies. This effect suggests a difference of parameters A_0_ and U_0_ in the previous pre-neuron stimulation experiment at constant frequency that was not accessible due to large variability in the switching characteristics from device to device (i.e. difference of A_0_ and U_0_ between 2 and 5 kHz was hindered by variability) while this difference is emphasized during STDP experiments. Nevertheless, qualitative description has been considered in the following for validating the proposed STDP implementation. A better description and understanding of the large variability in ECM devices should consider refinement of the model based on a more physical approach of switching modeling.

### Temperature effects as short time scale interactions

**Figure 3:**
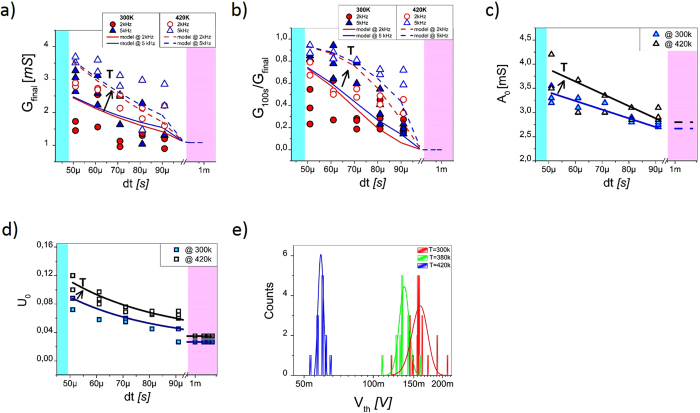
(**a**) Evolution of the threshold voltage for ON switching in conventional sweeping experiments (equivalent to Fig. 1a) and effect of temperature. (**b**) and (**c**) Potentiation and LTP-induced characteristic obtained for STDP measurements at 300 K and 420 K. Lines are model V2 calculation for 2 kHz (red) and 5 kHz (blue). (**d**) and (**e**) Evolution of fitting parameters U_0_ and A_0_ for STDP experiments at 300 K (filled symbols) and 420 K (open symbols). Lines are the exponential and linear fitting for U_0_ and A_0_, respectively.

The origin of the STDP feature could be mainly explained by two physical effects reported in ECM memory devices. The first one is the non-linear conductance relaxation in filamentary devices that was recently proposed by Du *et al*.^24^. In this oxide-based memory device, a second order memristor in which two state variables presented different relaxation time constant was used to attribute short term and long term effects to internal ionic dynamics. This model was able to describe both short term plasticity and STDP measurements without pulse overlapping. Following a similar approach, we performed measurements of conductance relaxation in time from 500 ns to 100 s. Different regimes of relaxation were not observed in the short time scale window and were a first indication of other effects involved in short time scale interactions between two successive pulses. A second effect that could reasonably explain the short time scale interaction is based on heating effects and subsequent heat dissipation after switching. A second pulse following a prior impulse can benefit from local heating in the switching region of the filament that increases the effect of this second excitation on potentiation. In order to evaluate the temperature effects in ECM cells, we performed STDP measurements while the sample was heated at 420 K. Resulting STDP measurements are presented in Fig. 3b and c. A clear shift of both potentiation and long term plasticity induction was measured with respect to room temperature measurements.

Fitting of the STDP measurements at 420 K with model V2 was possible by increasing A_0_ (a = 5.88 mS and m = −35 S/s in Fig. 3d) and U_0_ (u_a_ = 2.7 × 10^−2^, u_b_ = 0.45 and τ_T_ = 37.3 μs in Fig. 3e) dependency with dt. Thus, for fixed pulse amplitude, the increase in T corresponds to an increase of U_0_, the amount of potentiation induced by a given pulse, and of A_0_, the maximum synaptic conductance. For STDP measurements realized at 300 K, the increase of U_0_ and A_0_ when dt was decreased is a possible signature of higher local temperature when a prior pulse was applied shortly before the second one. If this experiment is not sufficient to attribute short-time scale interaction between pulses to heating effects only, it is a strong indication in favor of this possibility.

A second temperature analysis was realized by considering the evolution of the switching threshold (V_th_) during conventional sweeping measurements. V_th_ corresponds to the sharp jump in conductance observed in the IV sweeping measurements during SET process (Fig. 1a). By increasing the temperature from 300 K to 420 K, a clear decrease of V_th_ is obtained (Fig. 3a). Since pulse amplitude is fixed in our experiment, higher temperature result in switching pulses much larger than Vth thus resulting in larger potentiation. This trend is consistent with the evolution of U_0_ observed during short time scale interactions between two successive pulses where temperature effects are expected to play a significant role in switching. At the opposite, increasing T results in an increase of the volatility (Supplementary Figure S2) which is only effective on short time scale duration. To become fully consistent, refinement of our model should include two regimes of relaxation but the general behavior captured by a single characteristic time constant was a reasonable approximation.

### Interplay between different synaptic plasticity processes for learning

**Figure 4:**
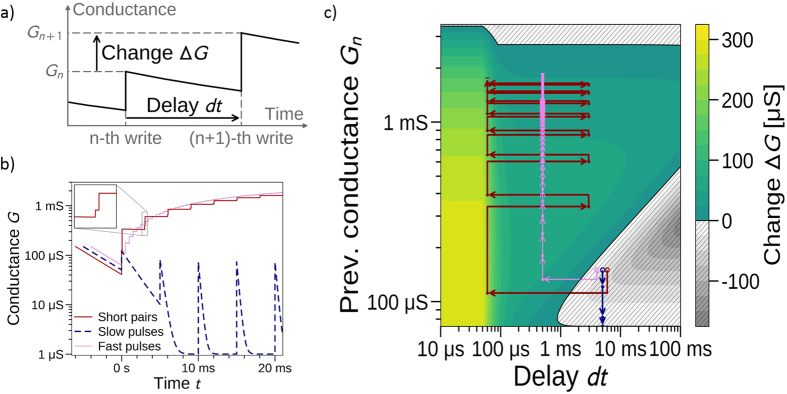
(**a**) Sketch of the variables used to describe the impact of a pair of (identical) programming pulses on the conductance of an ECM cell. (**b**) Evolution of the conductance with respect to time, with three different programming schemes: i) STDP potentiation (red solid line), ii) single programming events at low frequency (light blue dashed line) and (iii) single programming events at high frequency (pink thin line). The top left inset is a zoom on one of the double conductance jumps due to a STDP pair. (**c**) Landscape map of conductance change with respect to delay and previous value of conductance. Colors indicate the value of ΔG: the hatched gray areas correspond to negative ΔG, while the areas colored in green to yellow correspond to positive ΔG. The black solid line separating the hatched region from the region without hatches is a null contour. The arrows represent the jumps of conductance that the ECM cell experiences under the programming schemes of (**b**). Red arrows correspond to a train of STDP pairs that successfully bring the device to its high conductance state and LTP regime. Dashed light blue arrows correspond to regular single pulses at low frequency that keep the device in its low conductance state. Pink arrows correspond to regular pulse train at high frequency that potentiates the device toward its high conductance state and LTP regime.

Model V2 captures both pre-neuron potentiation (i.e. STP and short term to long term plasticity transition) obtained at dt > 200 μs and STDP interaction between equivalent pre and post pulses at dt < 200 μs. This model implicitely captures the thermal effects originating the STDP window. In order to highlight how an interplay between the different plastic features embedded in our devices can be used for computing in spike based systems, we first focus on the way an ECM cell reacts when two successive pulses are applied on it. Two spiking events (pre/pre or pre/post, as both pulses are equivalent) results in a change of conductance ΔG = G_n+1_ − G_n_ (Fig. 4a) that is positive or negative depending on the delay dt between pulses and on the conductance value G_n_ after the first pulse. Figure 4c shows a landscape plot of the conductance change ΔG with respect to these two parameters. Constant frequency programming events correspond to moving along a vertical path in this map while horizontal displacement corresponds to modification of the time interval between two consecutive programming spikes. Such map provides a comprehensive way of understanding the learning process of an ECM synapse. We can distinguish three main regions:

#### Region 1

for 0 < dt < 100 μs, yellow or light green area. This region corresponds to the highest values of ΔG. Such short delays between programming pulses corresponds to STDP interaction and result in conductance jumps that are larger than anywhere else in the landscape. Furthermore, as ΔG is positive everywhere, any programming pulse leads to potentiation.

#### Region 2

for dt > 100 μs, darker green area. ΔG is also positive everywhere and any spike effectively potentiates the synapse too. However, the amplitude of ΔG is significantly smaller than in the previous region: more programming events are required to reach a given value of conductance. This is due to the fact that the delay between the programming pulses is long enough for the parameters A_0_ and U_0_ to decay close to their asymptotic values: this pulse do not benefit from the STDP dynamic.

#### Region 3

for dt > 1 ms, grey with hatches area. The device stimulated by a pulse will experience a decrease of conductance (with respect to the previous spike) due to unsufficient potentiation and larger natural relaxation. A sudden decrease of frequency can bring a synapse in this region after a weak potentiation and results in relaxation of the conductance state. If a non-potentiated synapse is stimulated with low frequency pulses (i.e. dt > 1 ms), it will stay in its minimal conductance state corresponding to the lower boundary of this region (ΔG = 0). Upper boundary of this region (ΔG = 0) are unstable states that can either potentiate or depress if dt is slightly modified.

In order to potentiate a synapse with a G_n_ value equal to the minimal conductance (i.e. G_n_ = 70 μS) and to eventually induce long term plasticity and strong potentiation, events with dt < 1 ms need to be apply. Such a situation can happen because of an increase of the input event frequency or due to the presence of STDP pre/post pairs.

The arrows in Fig. 4c illustrate the previous statement. All three series of arrows correspond to cases starting from a G_n_ value of 150 μS (slightly potentiated synapse). The blue arrows are for a 5 ms-periodic train of spikes (low frequency stimulation). The effect of the first pulse corresponds to a decrease of conductance (with respect to the initial state). Next pulses keep the device in the low conductance state with no change in conductance from pulse to pulse. Delay between the programming events is long enough for the conductance to relax before each new event. Red arrows correspond to a synapse experiencing STDP events with short dt and leading to a strongly potentiated state. After a decrease of conductance between the initial state and the first pulse, the device experiences a short dt of 60 μs corresponding to a STDP event (second pulse) and inducing a strong change of conductance. A slow 3 ms-periodic repetition of few STDP pairs of pulses induces a strong potentiation. This path corresponds to short term to long term transition induced by STDP events. The pink arrows correspond to a potentiation induced by constant high frequency stimulation without STDP event (0.5 ms-periodic pulse stream). After a decrease of conductance between the initial state and the first pulse, the device experiences a continuous increase of conductance. Since potentiation induced by each pulse is weak, strongly potentiated state required a large number of events (with respect to STDP events). This path can eventually corresponds to short term to long term transition induced by pre-neuron only stimulation. Here, we consider only pulses that induce potentiation (i.e. positive polarity w.r.t. top electrode). Pulses of opposite polarity can in principle induce depression of the weight and allow for higher flexibility. In the particular case of our ECM cells, we observed a very strong depression with a single pulse that cancel any gradual learning. Using natural relaxation is consequently more convenient to implement an evolution of the synapse across multiple resistive states.

In order to evaluate how practical ECM cells that feature both STDP and short term to long term plasticity transition can be, we performed simulations of a simple recognition problem with a system using synapses that are based on model V2 described previously. The recognition problem is composed of vertically moving objects at fixed speed and direction but with random position in the three different lanes. This problem is a simplified version of a widely studied video of vehicles moving on a highway^33^.

**Figure 5:**
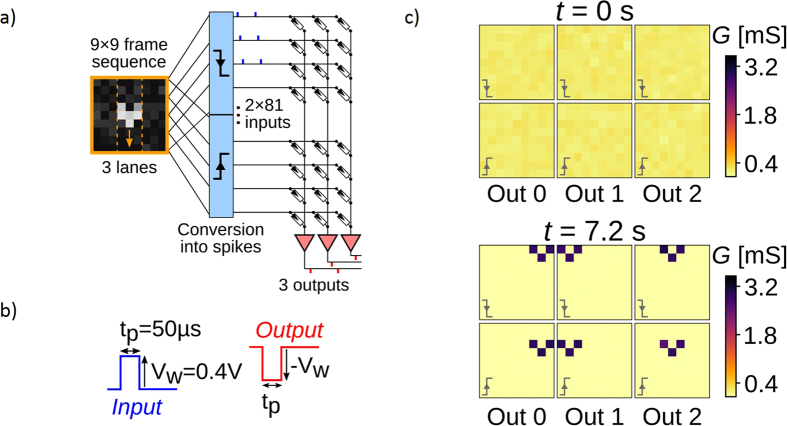
(**a**) Schematic of the system-level simulation workflow. The grayscale picture is an example of the frames used as raw input for the simulations. The levels of gray code the intensity of the pixels. Symbols in the blue blocks indicate the type of detection that is used to generate spikes from a sequence of these frames at the pre-neuron level. The upper, resp. lower, symbol corresponds to pre-neuron sensitive to a decrease, resp. an increase, of the pixel intensity. The three red triangles are the three output neurons. (**b**) Sketches of the spike waveforms used by the simulator for input (blue) and output (red) programming events of the ECM synapses. The baseline of both sketches corresponds to the ground. (**c**) The six conductance maps of the whole system, just before starting to apply input (programming) events (t = 0 s), and just after the last input event (t = 7.2 s). The label “Out 0/1/2” refers to the output neuron, and the small gray symbol bottom left of each conductance map indicates the type of input neurons these synapses are connected to (see (a)). The color of each pixel indicates the value of the conductance G for a given synapse.

The input neurons (or pre-neurons) present the video to the system, in a way inspired by a biological retina. Each pixel is associated with two neurons, one with sensitivity to increasing intensity, the second with sensitivity to decreasing intensity. An input neuron emits a spike when a change of pixel intensity over (below) an arbitrary threshold is detected (Fig. 5a, more details are given in supplementary info). These input neurons are connected to three output neurons (or post-neurons) via single ECM devices organized in a crossbar configuration. The crossbar circuit do not take into account wires’ resistance contribution. Note that sneak paths currents are not critical in this system since output neurons implement a virtual ground and signal is sent in parallel. When an output neuron is activated, it generates an output spike and a similar feedback spike is sent backward to the ECM devices (Fig. 5b). Such feedback operation can be realized through simple current conveyor^34^. In addition, it resets the two other output neurons and inhibits them during a short time window (more details are given in supplementary info). The total number of ECM devices is then 2 × 81 × 3.

ECM synapses learn using the two different dynamics that they feature: the STDP behavior and the short term to long term plasticity transition. Because input and output spikes have exact opposite waveforms, the learning rule is fully driven by the relative timings of the spikes received by the synapses. This embedded learning rule is therefore especially interesting from the CMOS circuitry point of view: the programming events have simple waveforms, and no external control circuit but neurons are required to implement the learning rule, contrary to systems that use other types of memristors^35^,^36^.

The two sets of 6 images in Fig. 5c plot the conductance maps of the ECM crossbar, before any input spike is applied (t = 0 s), and after the last input spike has been applied (t = 7.2 s). The color of one pixel in these maps represents the conductance value of the ECM synapse connected to a given pair of input and output neurons. The initial state of the synapses is set to a slightly potentiated conductance value (mean of 0.2 mS, with 16% of relative standard variation). One can observe the result of a successful learning: each output neuron specializes into a dedicated lane evidenced by clean patterns of three highly potentiated ECM cells in each conductance map. The patterns learnt in the three upper and the three lower conductance maps correspond respectively to the rear and to the front edge of the moving objects in the input video. This suggests that the learning reacts to short timescale correlations between inputs and output events. Since input spikes delays are bounded to be always longer than 1 ms (with exception of noise events) and initial states are only slightly potentiated (mean of 0.2 mS), the synapses will relax if they only experience input spikes (ΔG will fall in the grey hatched area, region 3 in Fig. 4c). Contrary to other memristive devices, depressing our ECM cells is not achieved with pulse overlapping of a dedicated type of pulses. Instead, we exploit the exponential relaxation of the devices in the short term plasticity regime between two pulses to achieve synaptic depression. This relaxation is driven by the τ_fac_ parameter of model V2 that depends on the conductance value of the ECM synapse: a device with a small conductance value will relax faster than a device with a larger conductance value. Only input spikes forming short delay with output spikes will trigger the learning process through the STDP dynamics that will eventually fully potentiate synapses into their LTP regime.

**Figure 6:**
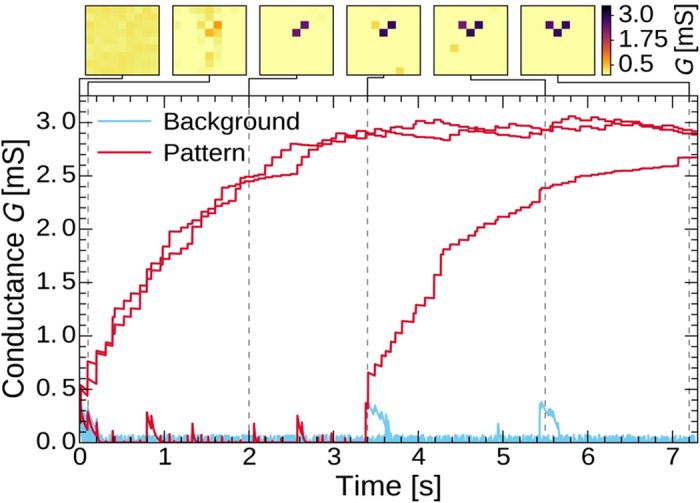
Evolution of the conductance G of the 81 ECM cells connected to one output neuron and to the input neurons that detect an increase of intensity (see Fig. 5). Red curves correspond to synapses that belong to the pattern that is learnt, and the light blue lines to all the other synapses, referred to as the “background”. Top pictures are conductance maps of the 81 studied synapses, plotted at six different instants indicated by gray vertical dashed lines.

Figure 6 depicts the evolution of the conductance of 81 synapses belonging to a conductance map of one output neuron that has successfully specialized in the central lane after presentation of 90 moving objects. The resulting learnt pattern is formed by the three synapses that are highly potentiated at the end of the simulation (dark purple in the top right conductance map snapshot). Their conductance curves are plotted in red color, while all the other synapses are designated as background, and plotted with light blue curves. At 0 s, the conductance values are normally distributed with a mean of 0.2 mS and a coefficient of variation of 16%, and each synapse is assumed to have been programmed 80 ms before. Between 0 s and 7.2 s, input spikes are presented to the synapses. During the first hundreds of milliseconds, all synapses but two relax. A partial pattern starts being learnt through input/output spike pairs applied on these two synapses that increase their conductance. Both STDP and non-STDP pairs are involved in the learning (see supplementary info). The STDP part of the model is essential to the learning process: when model V2 was substituted with model V1 (removing the STDP dynamics at short timescales), all synapses relax and no learning was observed. During the first three seconds, the third synapse with low conductance experiences only short term plasticity, and does not overcome the negative ΔG region. Around 3.4 s, it is finally programmed by two close STDP pairs, triggering its short term to long term plasticity transition. Additional input/output spike pairs, with STDP and non-STDP dynamics, achieve to potentiate this synapse, and thus the learning of the complete pattern. Around 3.4 s and 5.4 s, two synapses of the background are wrongly potentiated, appearing in darker yellow in the two corresponding conductance map snapshots. Since input spike delay is always set to be larger than 1 ms, these accidental potentiations are due to noise that creates non-meaningful pairs of spikes. As these noisy events are rare, contrary to the input/output pairs produced by the learning of the pattern, they do not trigger the short term to long term plasticity transition and the synapses are depressed through their natural relaxation. Thus, once the background has relaxed, this system is rather resilient to low frequency noise events. Compared to noiseless simulations, where at least two patterns are cleanly learnt after 93% of the runs, the system still achieves a performance of 70% when some noise is added to the input events (additional details about the noise in supporting info), as indicated in Table 1. One qualifies as “clean” a conductance pattern with exactly 2 × 3 saturated synapses that form a shape similar to the ones in Fig. 5c.

Another constant concern about a system with nanodevices is how device variability impacts its performance. To evaluate this, we performed simulations with variability added on a few key parameters of the model (details are provided in the supplementary info). The preliminary results show that the system is noticeably resilient to such imperfections. For example, the Table 1 shows that it still achieves a performance of 85% in the case of noiseless inputs. If we consider runs where all three patterns are learnt, performance is 43.3% without device variability and 38.3% with device variability.

## Conclusions

We demonstrated in this paper long term plasticity induction by STDP. As learning in neural networks is mostly associated to Hebbian-type plasticity, we implemented a bio-realistic protocol in order to demonstrate Hebbian STDP corresponding to an increase of potentiation when correlated events (i.e. spiking) between pre and post neurons are detected. Not only potentiation was increased during STDP events but also the long term plasticity characteristic of the synaptic weight modification. STDP was described in terms of heating effect during switching which naturally implement pulse timing correlations. Combination of both short term to long term plasticity transition and STDP-induced long term plasticity in the same memory device is highly valuable since it offers the possibility to deal with rate coding strategies as in the case of BCM concept^37^ and with temporal coding approaches where meaningful information are encoded through the precise timing of neurons. We showed a simple recognition task and demonstrated dynamic motion learning based on the proposed mechanisms. Such results are a proof-of-concept of the promising impact of the interplay between different synaptic plasticity mechanisms (i.e. STDP and short term to long term transition) at synaptic level. This approach represents a key point to exploit biological neural networks efficiency for future neuromorphic systems.

## Methods

Electro Chemical Metallization cells were fabricated in a crosspoint configuration. Bottom electrodes were patterned by e-beam lithography and deposited by metal evaporation of 5 nm Ti and 30 nm Ag. A 60 nm Ag_2_S was thermally evaporated from pure Ag_2_S material in secondary vacuum conditions. Thickness was check by quartz balance. Pt top electrode were patterned by e-beam lithography and deposited by metal evaporation.

Electrical measurements were performed on a B1500 Agilent parameter analyzer with B1530 option for ultrafast I-V measurements and pulse generation. The STDP protocol was automatized via Visual Studio software. All measurements were performed in ambient conditions with a standard probe station setup.

## Additional Information

**How to cite this article**: La Barbera, S. *et al*. Interplay of multiple synaptic plasticity features in filamentary memristive devices for neuromorphic computing. *Sci. Rep.* **6**, 39216; doi: 10.1038/srep39216 (2016).

**Publisher's note:** Springer Nature remains neutral with regard to jurisdictional claims in published maps and institutional affiliations.

## Supplementary Material

Supplementary Information

## Figures and Tables

**Table 1:** Proportion of simulations where at least two of the three patterns have been cleanly learnt.

	Noiseless inputs	Noisy inputs
Without device variability	93.3%	70%
With device variability	85%	60%

A total of 120 and 60 simulations were performed, respectively with and without device variability (whether or not the inputs were noisy).
