# Supplementary material for: Interplay of multiple synaptic plasticity features in filamentary memristive devices for neuromorphic computing
Source: Sci Rep. 2016 Dec 16;6:39216. doi: 10.1038/srep39216 (PMC5159796; doi:10.1038/srep39216)
Supplement: Supplementary Information [file srep39216-s1.pdf]

SUPPLEMENTARY MATERIALS

Interplay of multiple synaptic plasticity features in filamentary memristive devices for neuromorphic computing

Selina La Barbera [1,§], Adrien F. Vincent [2,§], Dominique Vuillaume [1], Damien Querlioz [2] and Fabien Alibart[1,*]

[1] : Institut of Electronic, Microelectronic and Nanoelectronic, CNRS, boulevard Poincarré CS 60069, 59652 Villeneuve d'Ascq, France

[2] : Centre de Nanosciences et de Nanotechnologies, CNRS, Univ. Paris-Sud, Université Paris-Saclay, C2N - Orsay, 91405 Orsay cedex, France

[§] : these two authors contributed equally to this work

[*] : corresponding author: fabien.alibart@iemn.univ-lille1.fr

A/ Supplementary experimental data

- **Experimental extraction of the characteristic time constant during pulse induce potentiation**

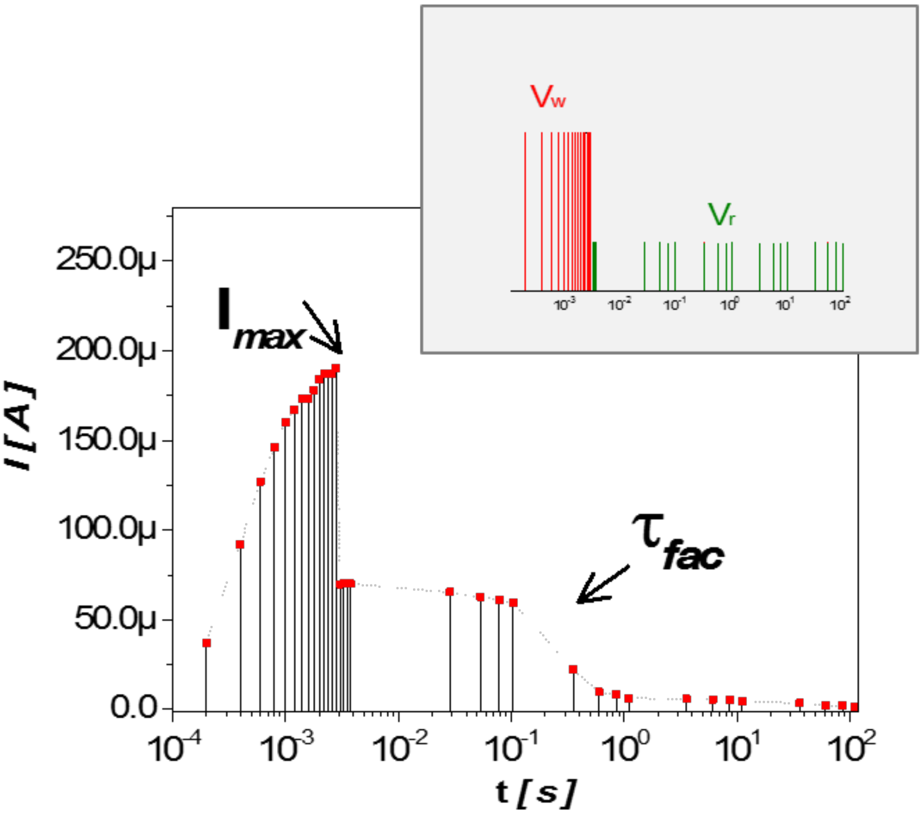

Figure S1: Experimental extraction of the characteristic relaxation time constant. A burst of potentiating pulses V_w is applied and followed by a sampling of conductance over 6 decades of time with short non-disturbing pulses V_r.

- Effect of temperature on the relaxation of ECM cells

We report on figure S2 the evolution of G100s/Gmax as a function of Gmax. Since characteristic time constant of relaxation τ is directly related to G100s/Gmax value, we can evaluate the effect of temperature on relaxation. For the same Gmax value, τ decrease when temperature is increased (i.e. G100s/Gmax decrease) as indicated by the red arrow.

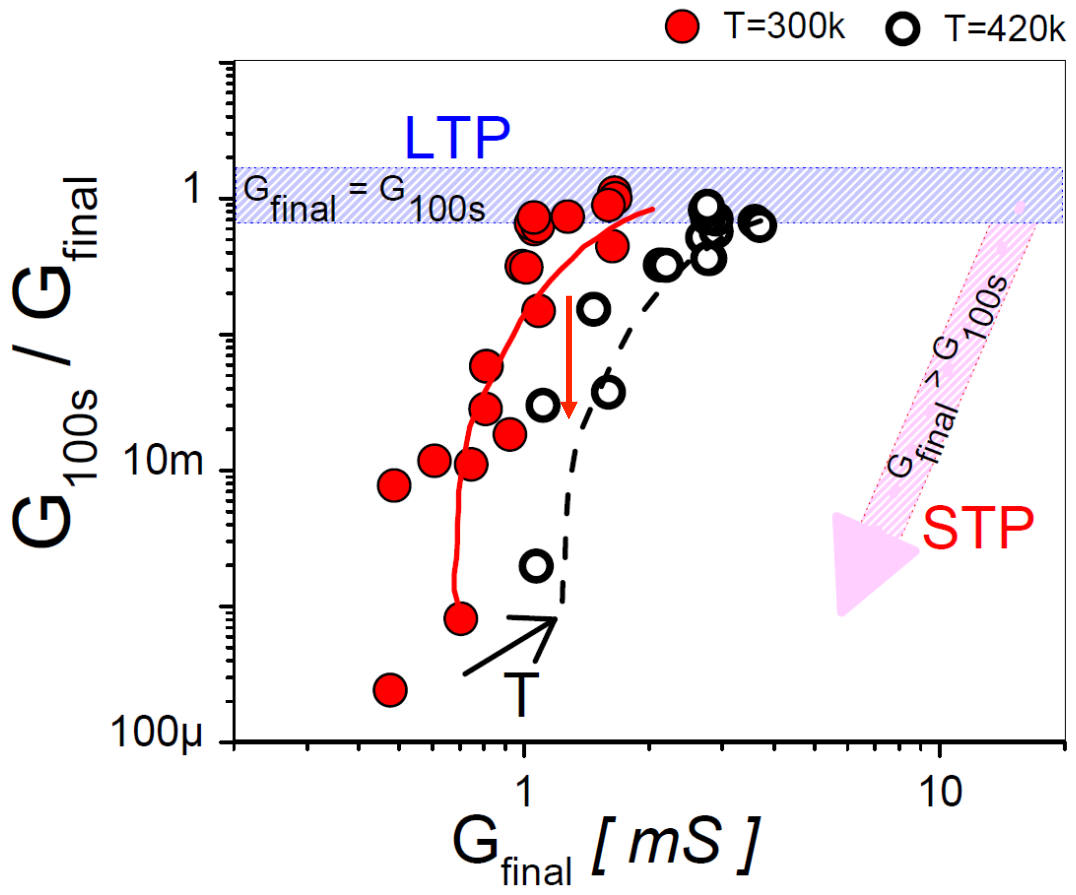

Figure S2: effect of temperature on G100s/Gmax. The two measurements have been realized at T=300K (red dots) and 420K (black circles)

B/ Methods of the system-level simulations

1 -- Input video

The video used to generate the input spikes is composed of 9x9 frames. These frames represent three non-overlapping vertical lanes with equal width. On these lanes, 6-pixel patterns are going along, from the top to the bottom. These patterns have all the same shape, speed and direction. There is never more than one single pattern on a frame.

An input video is 7.2 s-long, with 90 successive cycles of 80 ms. During each cycle, three patterns are presented– one on each lane– in random order. This is done to ensure a minimal spike activity on each synapse during the learning step. In the absence of noise, the minimal (maximal) delay between two input spikes on the same synapse is about 27 ms (133 ms).

The framerate of the input video is 450 FPS. A pattern goes across the window formed by the frames in 12 frames, activating any pixel it goes over. Without any noise, the pixels that are activated by a pattern have all the same value of one (in arbitrary units), while all the other pixels have a null value.

Noise can added to the input video, before the conversion into spikes. To do this, each pixel has a probability Pr(0→1|0)to switch off if it is active, and a probability Pr(1→0|1) to activate if it is currently inactive. This noise is independently and identically distributed on all the pixels of each frame.

The video attached to this supplementary material presents one typical sequence used for our system-level simulations. For the sake of clarity, the framerate of the video has been slowed down thirty times compared to real-time, from 450 FPS to 15 FPS

2 – Conversion into spikes

The input video sequence is converted into spikes by detecting changes of the pixel intensity between two successive frames. This is reminiscent of the bio-inspired vision sensor [1], in a largely simplified fashion. Every single pixel of the 81 pixels constituting a frame is associated with two input spiking neurons. In each one of these 81 pairs of neurons, one neuron emits a spike when it detects an increase of intensity beyond a threshold T^+, while the other does the same for a decrease of intensity below a threshold T^-. In our simulations, $T^+ = T^- = 0.5$ for all the input neurons. Besides a refractory period prevents an input neuron from spiking during 1 ms after it emitted a previous spike.

Considering two successive frames k and k+1, the exact instant a spike is emitted by an input neuron due to a sufficient change of intensity between these two frames is uniformly drawn within a 1 ms-long window just before the frame k+1 starts.

The noise added into the input video typically corresponds in our simulations to an average of 2 supplementary spikes and 0.1 missing spike for 100 correct spikes.

3 – Output neurons

Outputs are Leaky-Integrate-and-Fire (LIF) neurons. In our simulator, they are functionally implemented as simple leaky integrators that emit spikes when they have integrated currents up to a given threshold. After having spiked, an output neuron is reset during a refractory period of 20 ms. Furthermore, it resets all the other output neurons too, during a period of 15 ms. This lateral inhibition prevents the output neurons from learning the exact same pattern.

During the learning phase, due to the inhibition between output neurons, if two vehicles appear at the same time, one of the vehicle will be ignored. After learning, however, lateral inhibition becomes unnecessary and can be deactivated. The system can then recognize two vehicles arriving at the same time.

B/ Additional discussions

1 – Other remarkable paths in the ΔG landscape

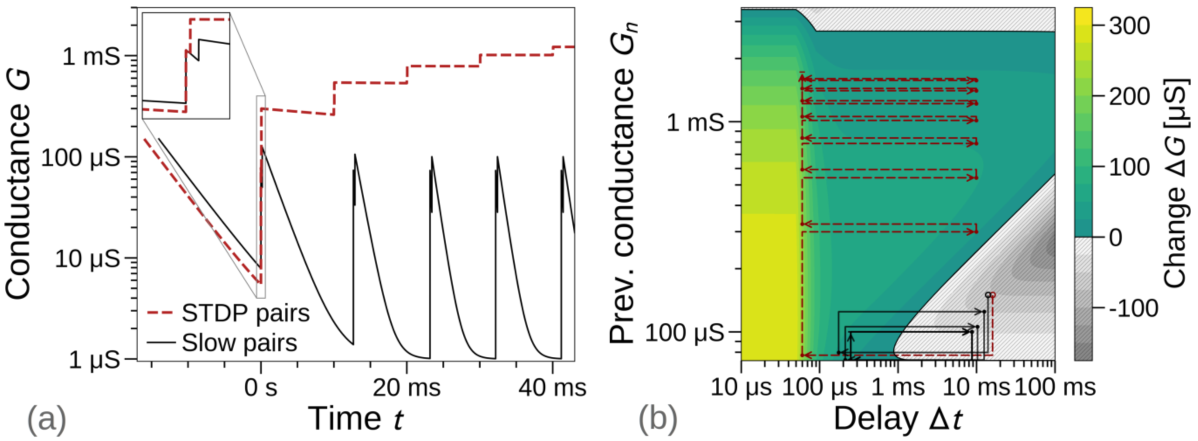

Figure S3: ***(a)*** **Evolution of the conductance with respect to the time, and** ***(b)*** **A landscape map of conductance change ΔG with respect to the delay** ***dt*** **and the previous value of conductance G_n. The arrows represent the jumps of conductance that the ECM cell experiences under the programming schemes of (a). The unfilled circles depicts the initial conditions. Two different schemes of stream of programming pulse pairs are depicted, with the same line style in both panels. i) The dark red dashed lines depicts a case similar to the one in solid red lines in figure 4b in the article, where the pulses of a pair are close enough to excite the STDP dynamic of the ECM cell: the synapse experiences the short-term to long-term plasticity transition and goes toward its highest conductance state. ii) The solid black lines corresponds to a case where the delay between the pulses of a pair is too long and does not excite the STDP behavior: from an initial state similar to (i), the ECM cell falls into a limit cycle after a few pairs and is not long-term potentiated.**

Fig. S3presents (a) the evolution of the conductance, as well as (b) the travel in the ΔG landscape, that are associated with two programming schemes that use pairs of pulses.

Dashed dark red plots depict the case of a regular train of programming STDP pairs, reminiscent of the case plotted in solid dark red in Fig. 4b of the article, with a similar impact on the synapse. Black solid lines present the case of a pair stream with delay between the pulses of a pair about 0.25 ms, which is too slow to excite the STDP behavior. In this case, the synapse stays in the short-term plasticity regime, oscillating between two levels in the ΔG landscape, without ever going toward the saturation. This is a simplistic view of the situation experienced by the synapses that output spikes excite in a fashion which is not highly correlated with the inputs they are connected to.

During a realistic simulation with noise and shuffled input patterns, the conductance of a synapse evolves in a significantly more complicated way than the four extreme schemes cases that one has presented. In particular, a combination of several of these extreme schemes (as other ones) can be observed for the synapses that learn a pattern.

2 – Interplay between STDP and non-STDP programming pairs

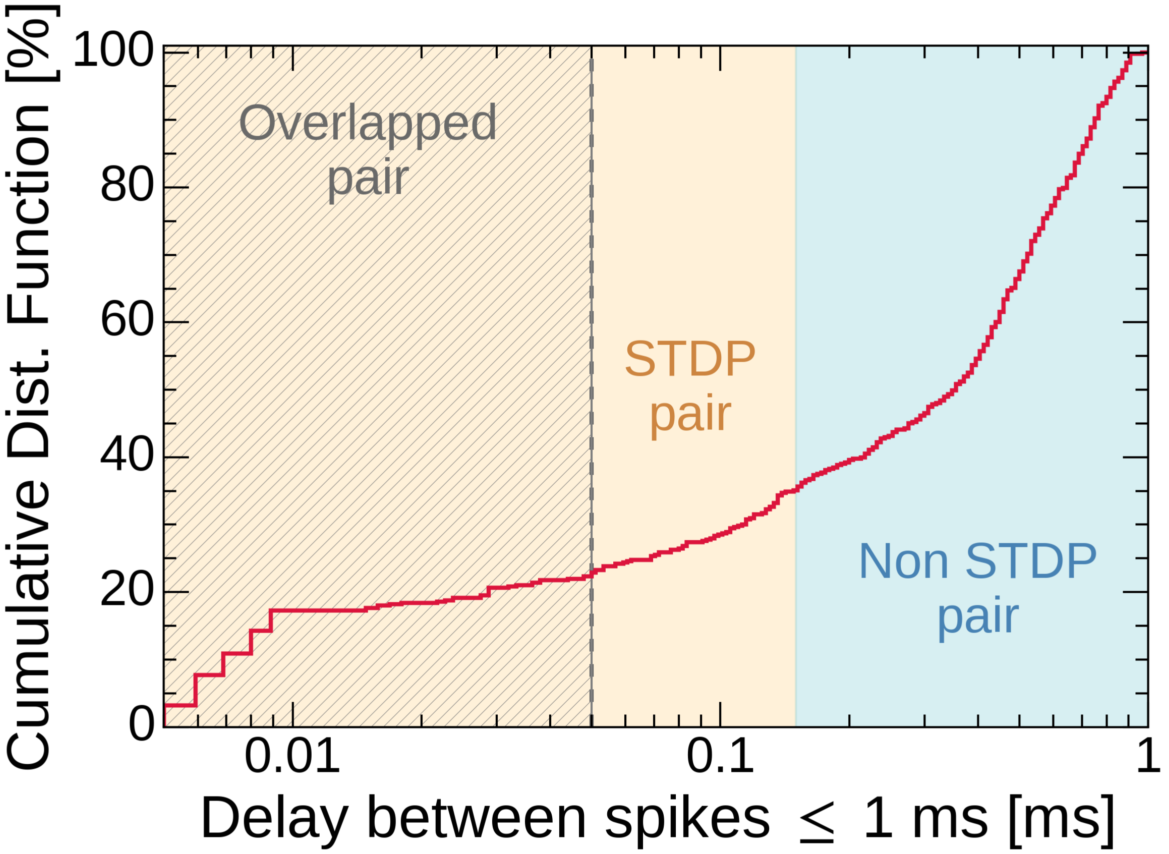

Figure S4: The red solid line is the empirical cumulative distribution function (CDF) of delays that are shorter than 1 ms, in a case where all three patterns have been learnt. The delays used to estimate the CDF correspond to all the delays between all the spikes that have applied on the 3*162 synapses of our system during the learning. The hatched region depicts a delay that is shorter that the duration of a programming pulse. Orange and blue regions respectively indicate if a delay excite or not the STDP dynamics of the ECM device. The value of 150 µs for the boundary is based on the observation of the ΔG landscape in Fig. S3b.

Red curve in fig. S4 depicts the repartition of the programming event pairs with a delay below 1 ms, under the form of an empirical cumulative distribution function (CDF). This CDF has been computed on all the delays experienced by the 3*162 synapses during a particular simulation run. From the ΔG landscape, one knows that delays shorter than 1 ms always potentiate the synapse their applied to. Besides, as the input events have been designed not to excite such fast dynamics, and as the output neurons cannot spike twice in less than 1 ms, the delays accounted for to draw Fig. S4 are necessarily due to combinations of pre- and post-synaptic events. The values are smoothly distributed over the whole possible range: the synapses are stimulated with an interplay of the different dynamics they feature. About 1/3 of the pairs excite the STDP dynamics, and they are of utmost importance for the learning: if the STDP part of the synaptic model is replaced with its long-delay behavior, no learning is observed at all.

Furthermore, about 1/3 of these STDP pairs are not overlapped at all.

The rough steps for delays below 10 µs are mainly due to discretization of time in the simulation.

3 – Results with device variability

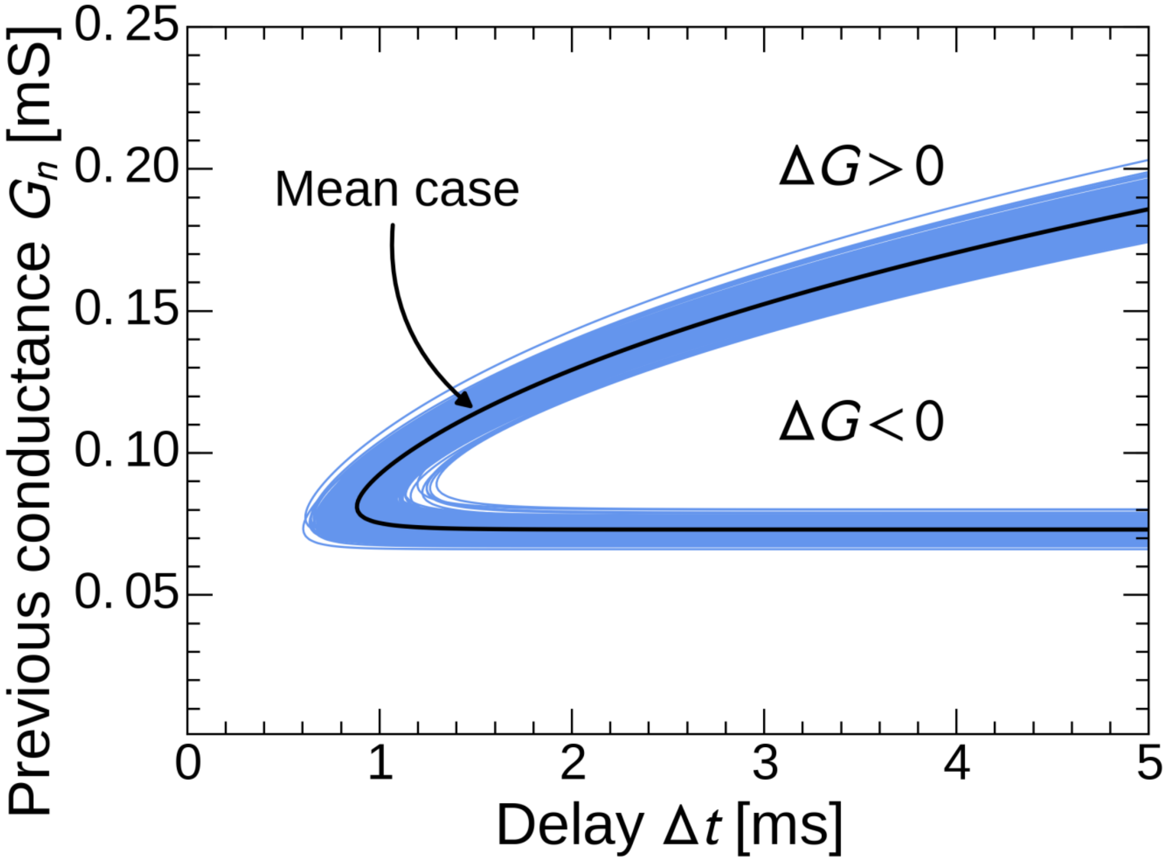

Figure S5: Contour plot of the change of conductance ΔG, with respect to the delay *dt* and the previous value of conductance G_n, showing the impact of device variability. Each thin blue solid line is the null contour associated with one of the 3*162 synapses used in a simulation testing the impact of variability. Gaussian dispersions with a coefficient of variation (CV) of 3 % are added on all the parameters in Eq. (2.1): u_a, u_b and τ_T, and a CV of 5 % on the prefactor a in the expression $\tau_{fac}(G)$.

To study the resilience to device variation of a learning-capable system that uses ECM synapses, we performed simulations where dispersion has been added to some of the parameters in the synaptic model. One added independent Gaussian dispersions on u_a, u_b and τ_T (Eq. 2.1), with a coefficient of variation of 3 %, as well as on the prefactor a in the expression $\tau_{fac}(G)$, with CV = 5 %. Fig. S5 shows how the null contour in the ΔG landscape is affected by such device variation. Every light blue line corresponds to one of the 3*162 synapses in the crossbar, and the thick black line is the null contour without variability. One can observe that the synapses have significantly different behavior from one another. Nevertheless, the first results given in the main article suggest that the system still achieves good performance, learning for example at least two clean patterns on three in 85 % of the simulation runs with noiseless inputs, to compare with 93 % without device variation.

1. Frame-free dynamic digital vision," Proceedings of Intl. Symp. on Secure-Life Electronics, Advanced Electronics for Quality Life and Society, 2008, pp. 21-26.
